# Modeling of X-ray pulse propagation in beamline systems using a 6D phase space ray-tracing method

**DOI:** 10.1107/S1600577525004849

**Published:** 2025-06-23

**Authors:** Kai Hu, Zhenjiang Xing, Chuan Yang, Ye Zhu, Chen Wu, Zhongmin Xu, Qiuping Wang, Weiqing Zhang

**Affiliations:** a National Synchrotron Radiation Laboratory University of Science and Technology of China Hefei Anhui230029 China; b Institute of Advanced Light Source Facilities, Shenzhen518107, China; c Southern University of Science and Technology Shenzhen Guangdong518055 China; d State Key Laboratory of Molecular Reaction Dynamics, Dalian Institute of Chemical Physics Chinese Academy of Sciences Dalian116023 China; SLAC National Accelerator Laboratory, USA

**Keywords:** X-ray pulse propagation, 6D phase spacing ray-tracing, beamline design, free electron laser, synchrotron radiation

## Abstract

With the rapid development of X-ray free-electron lasers (XFELs) that can generate ultrashort X-ray pulses with a duration range from attoseconds to femtoseconds, the study of ultrashort XFEL pulse propagation in beamline systems is increasingly important, especially in dispersive beamline systems. We developed a 6D phase space ray-tracing method to simulate pulse propagation in dispersive soft X-ray optical systems. We validated this method by simulating a typical dispersive optical system: a grating monochromator. The simulation indicated that the spatiotemporal properties such as pulse front tilt, pulse front rotation and angular dispersion can be described. Using this approach, we performed a start-to-end simulation of the Shenzhen Superconducting Soft X-ray Free Electron Laser (S^3^FEL) FEL-1 beamline. Compared with the 3D pulse propagation method based on Fourier optics, this significantly reduces the simulation time. Our work provides a useful tool for X-ray beamline systems design.

## Abstract 2

This work develops a 6D phase space tracing module in the *FURION* software. The 6D phase space tracing provides a solution for the rapid simulation of ultrashort pulse propagation in X-ray beamlines, especially in dispersive beamline systems.

## 1. Introduction

X-ray free-electron lasers (XFELs) have undergone rapid development with several facilities built or under construction around the world, including LCLS (Emma *et al.*, 2010 ▸), SACLA (Ishikawa *et al.*, 2012 ▸), PAL-XFEL (Kang *et al.*, 2017 ▸), SwissFEL (Prat *et al.*, 2020 ▸), European XFEL (Decking *et al.*, 2020 ▸) and SHINE (Zhao *et al.*, 2018 ▸). These facilities can generate ultra-short X-ray pulses, which provide a powerful tool to study the microscopic world at the atomic scale as well as ultra-fast dynamic processes. In the soft X-ray regime, the beamlines of XFEL facilities usually adopt grating-based dispersive optical devices, such as monochromators (Gerasimova *et al.*, 2022 ▸), spectrometers (Principi *et al.*, 2024 ▸), beam splitters (Liu *et al.*, 2010 ▸), compressors (Miotti *et al.*, 2017 ▸; Yang *et al.*, 2023 ▸) and so on. These dispersive optics introduce complex spatiotemporal coupling effects, significantly increasing the difficulty of numerical simulation. There is a significant demand for simulation tools that can analyze how dispersion and spatiotemporal coupling occur along XFEL beamline systems. In the synchrotron radiation light source and FEL community, several software packages have been developed for numerical simulations of beamline systems, including *SHADOW* (Welnak *et al.*, 1994 ▸), *SRW* (Chubar & Elleaume, 1998 ▸), *XRT* (Klementiev & Chernikov, 2014 ▸), *HYBRID* (Shi *et al.*, 2014 ▸), *MOI* (Meng *et al.*, 2015 ▸), *WPG* (Samoylova *et al.*, 2016 ▸) and *OPC* (Karssenberg *et al.*, 2006 ▸). These software tools have demonstrated excellent performance in describing the evolution of the transverse beam profile in non-dispersive beamline systems. However, they cannot calculate the longitudinal distribution evolution of ultra-short pulses in dispersive X-ray beamline systems, nor can they account for spatiotemporal coupling effects, such as pulse front tilt, pulse front rotation, pulse stretching, pulse compression and spatial chirp. These effects, arising in dispersive X-ray beamline systems, significantly increase the complexity of numerical simulations. Therefore, the numerical simulation of ultra-short pulse propagation in dispersive X-ray beamline systems is still a challenge.

To evaluate the propagation of ultra-short pulses in dispersive X-ray beamline systems, we established theoretical and numerical approaches in our previous research (Hu *et al.*, 2023*a* ▸; Hu *et al.*, 2023 ▸*b*; Zhu *et al.*, 2024 ▸). Kostenbauder matrices for X-ray optics have been studied in the literature (Hu *et al.*, 2023*a* ▸). An analytical solution for the spatiotemporal response of dispersive X-ray optics to ultra-short pulses was derived in recent work (Hu *et al.*, 2023*b* ▸). Based on Fourier optics, we developed the software package *FURION* that can estimate pulse propagation in dispersive X-ray systems (Zhu *et al.*, 2024 ▸). However, the speed of processing based on Fourier optics, especially in 3D pulse propagation, tends to be slow. In this work, we developed a 6D phase space ray-tracing module in *FURION*, which significantly reduces simulation time compared with the methods of Fourier optics. The paper is organized as follows: Section 2 introduces the geometric source in 6D phase space; Section 3 introduces the 6D phase space ray-tracing method through optical devices; Section 4 simulates the propagation of ultra-short pulses through a grating monochromator for benchmarking; Section 5 describes a start-to-end simulation of the FEL-1 beamline from the source point to the Spectroscopy and Coherent diffraction Imaging (SCI) endstation at S^3^FEL using the 6D phase space ray-tracing method.

## 2. Geometric source in 6D phase space

In this section, we introduce the model of the 6D geometric source. The geometric source module includes the following types: unchirped Gaussian sources, chirped Gaussian sources and geometric FEL sources.

For the 6D Gaussian geometric source, the intensity can be described by the probability distribution *P*(**V**) = 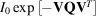 in the 6D phase space described by the vector **V** = (*x*, *y*, θ_*x*_, θ_*y*_, *t*, *v*). The matrix **Q** characterizes the coupling coefficients between different components in vector **V**, and can be expressed as 
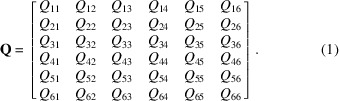
Here, we present an expression for a typical 6D Gaussian geometric source with temporal chirp,
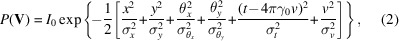
where σ_*x*_, σ_*y*_, 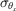, 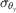, σ_*t*_ and σ_*v*_ are the root mean square (RMS) values of the source intensity in the six components of vector **V**. The parameter γ_0_ represents the chirp factor. An unchirped Gaussian source is indicated when the chirp factor γ_0_ equals zero. When γ_0_ is nonzero, the source is characterized as a chirped Gaussian source.

For the geometric FEL source, we transform the 3D FEL source generated by *GENESIS* (version 1.3; Reiche, 1999 ▸) into 6D geometric sources. The specific method is as follows: *GENESIS* generates a 3D optical field *E*(*x*, *y*, *t*), with the corresponding intensity distribution *I*(*x*, *y*, *t*). By performing a Fourier transform on *E*(*x*, *y*, *t*) we can obtain *E*(θ_*x*_, θ_*y*_, *v*) with the corresponding intensity distribution *I*(θ_*x*_, θ_*y*_, *v*). The probability distribution for the 6D geometric FEL source can be described by 
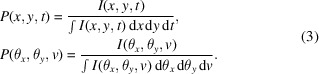


## 3. 6D ray-tracing through X-ray optics

The *FURION* module provides various types of X-ray optics, including toroidal gratings, spherical gratings, cylindrical gratings, planar gratings, varied-line-spacing (VLS) toroidal gratings, VLS spherical gratings, VLS cylindrical gratings, VLS planar gratings, toroidal mirrors, spherical mirrors, cylindrical mirrors, elliptical cylindrical mirrors, ellipsoidal mirrors, parabolic cylindrical mirrors, rotating parabolic mirrors and so on. In this section, we introduce 6D phase space ray-tracing through X-ray optics.

**Figure 1:**
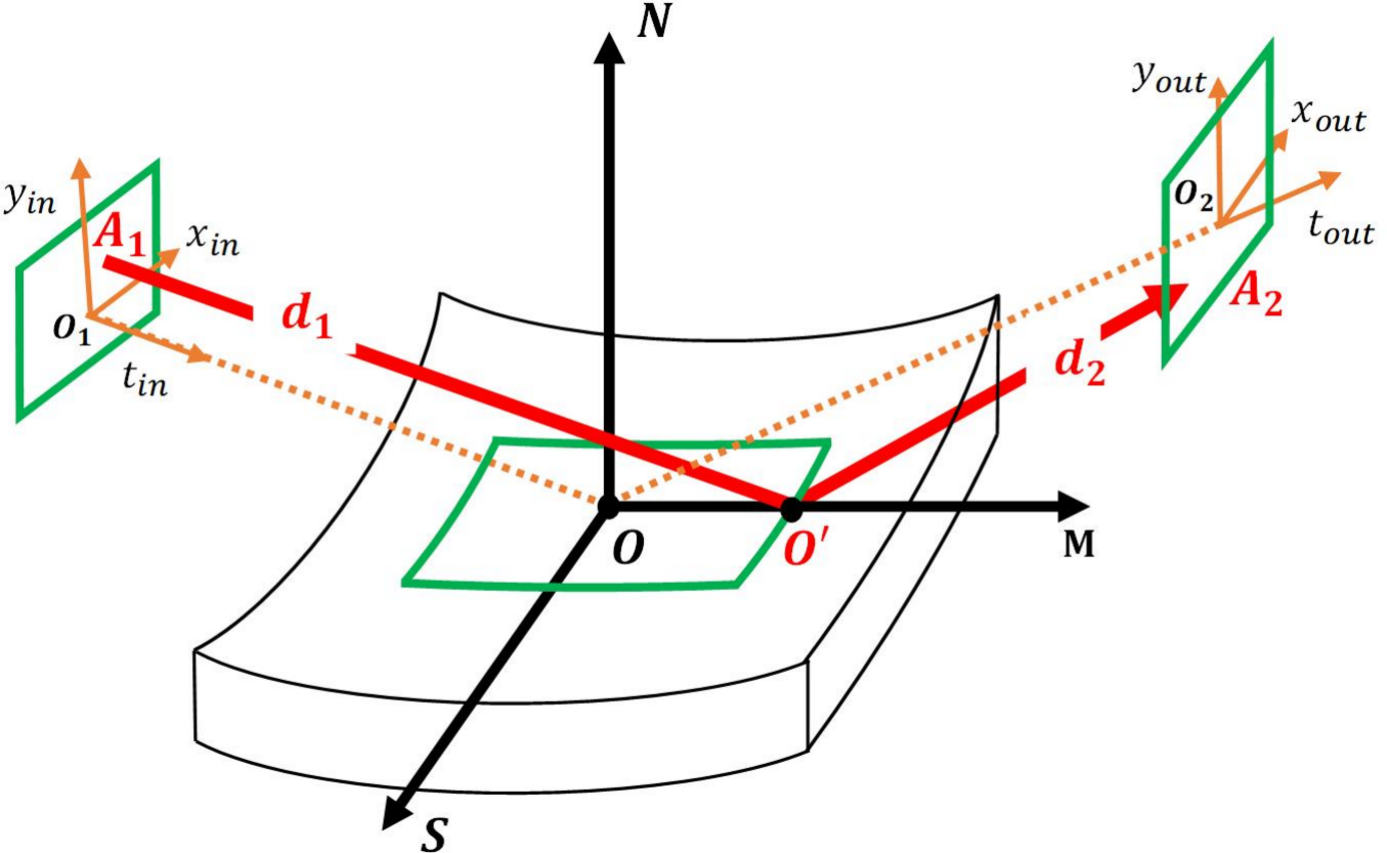
Schematic of the 6D phase space ray-tracing method.

In 6D phase space ray-tracing, it is essential to characterize the pulse in both transverse (beam size and divergence) and longitudinal (time and frequency) dimensions. As shown in Fig. 1 ▸, an upward optical device is positioned in the *O*–*S*–*N*–*M* coordinate system with source coordinate system *O*_1_–*x*_in_–*y*_in_–*t*_in_ and image coordinate system *O*_1_–*x*_in_–*y*_in_–*t*_in_. The primary optic axis is denoted *O*_1_–*O*–*O*_2_. An arbitrary ray is traced along the path *A*_1_–*O*′–*A*_2_. This ray can be described by vector **V** = (*x*, θ_*x*_, *y*, θ_*y*_, *t*, *v*). Here (*x*, θ_*x*_, *y*, θ_*y*_, *t*, *v*) represent the deviations from the central ray, which lies along the primary optical axis with the vector notation **V**_0_ = (0, 0, 0, 0, 0, 0).

The transverse components (*x*, θ_*x*_, *y*, θ_*y*_) can be obtained through direct ray-tracing. This method is commonly used in ray-tracing software, such as *SHADOW* and *XRT*, hence we will not provide a detailed introduction here. As for the longitudinal components (*t*, *v*), they can be described by 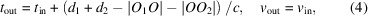
where *t*_out_ and *v*_out_ represent the time and frequency components after propagating through free space or optical devices. *t*_in_ and *v*_in_ denote the time and frequency components before propagating through free space or optical devices. The speed of light is denoted by *c*, while *d*_1_ and *d*_2_ correspond to the length of *A*_1_*O*′ and *O*′*A*_2_, respectively.

To compensate the diffraction effect caused by slits and optical devices with limited apertures, we extend the hybrid method (Shi *et al.*, 2014 ▸) to the 6D phase space ray-tracing model. The ray divergences are resampled after slits and limited aperture optics. This process aligns with the angular distribution derived from the Fraunhofer diffraction approximation.

## 4. Comparison and validation

In this section, we validate the 6D phase space ray-tracing method by simulating a typical X-ray system, a planar VLS grating monochromator system commonly adopted in soft X-ray beamline systems. We compare the propagation characteristics of ultra-short X-ray pulses through a VLS grating monochromator using the 6D phase space ray-tracing method with *SRW* (Chubar & Elleaume, 1998 ▸), *Shadow *(Welnak *et al.*, 1994 ▸), the **K**-matrix method (Hu *et al.*, 2023*a* ▸) and the analytic method (Zhu *et al.*, 2024 ▸). The VLS grating monochromator consists of a planar pre-mirror, a VLS grating and an exit slit. In the simulation, the distance from the source point to the VLS grating is 30 m, and the exit slit is located 15 m downstream of the VLS grating. The specific parameters of the source and the monochromator are detailed in Table 1 ▸.

**Figure 2:**
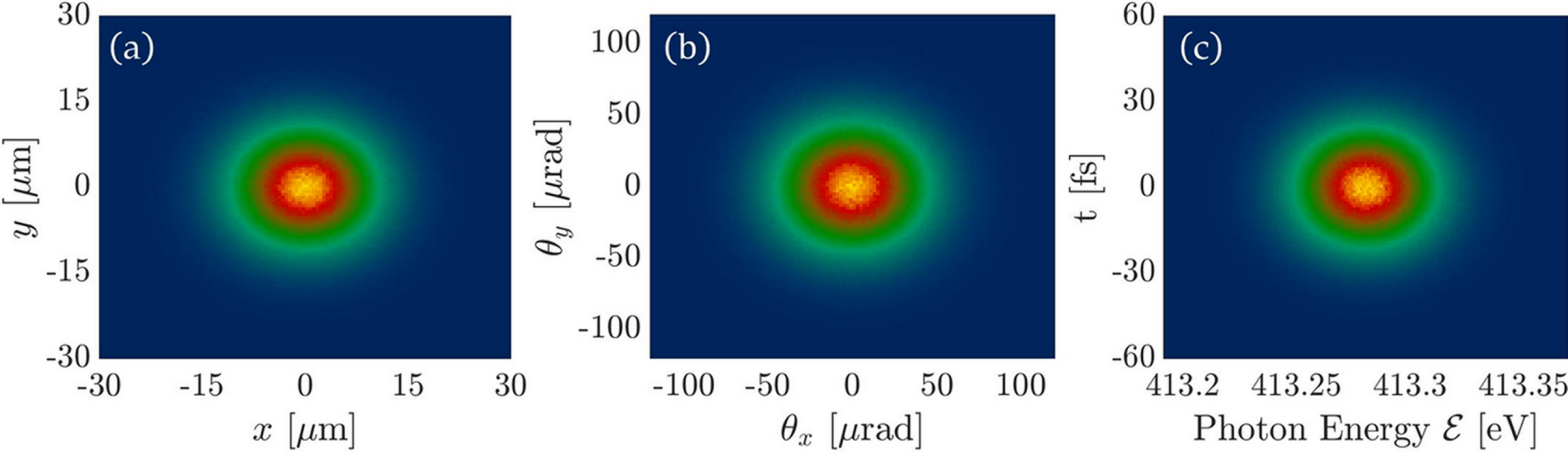
6D Gaussian geometric source generated by the statistics method. (*a*) Intensity projection in the (*x*, *y*) domain. (*b*) Intensity projection in the (θ_*x*_,θ_*y*_) domain. (*c*) Intensity projection in the 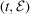 domain.

In the simulation, a Gaussian geometric source is employed. The central photon energy of the source is 413.3 eV, and both the beam size in the *x*, *y* dimensions are 7.56 µm. The source is a Fourier transform-limited pulse, with a pulse duration of 14.86 fs and a bandwidth of 0.022 eV. The RMS of the divergence is 31.58 µrad. The grating groove density is 150 lines mm^−1^ and the VLS parameter is 3.714 × 10^−4^ lines mm^−1^. The incident angle of the X-ray pulse on the grating is 88.124° and the diffraction angle is 87.450°. The Gaussian geometric source is generated using the statistical method introduced in Section 2. Figs. 2 ▸(*a*), 2 ▸(*b*) and 2 ▸(*c*) present the intensity distribution of the Gaussian geometric source in the (*x*, *y*), (θ_*x*_, θ_*y*_) and 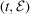 domains, respectively.

**Figure 3:**
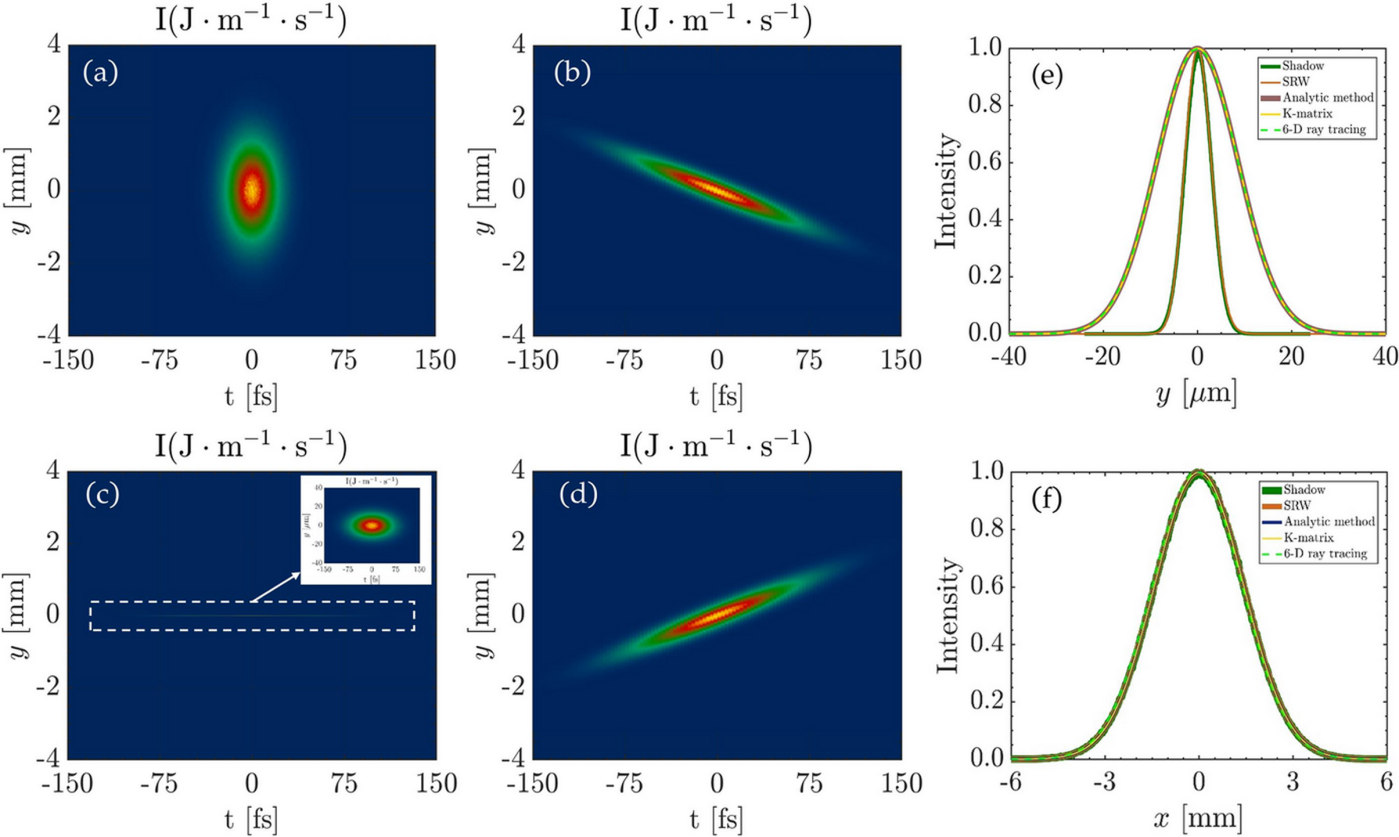
Intensity distribution in the (*y*, *t*) domain at different positions. (*a*) Before the VLS grating, σ_*y*_ and σ_*t*_ are 948.2 µm and 14.87 fs. (*b*) After propagating 7.5 m after the planar VLS grating, σ_*y*_ and σ_*t*_ are 643 µm and 45.95 fs. (*c*) After propagating 15 m after the planar VLS grating, at the focus (exit slit), σ_*y*_ and σ_*t*_ are 8.6 µm and 45.95 fs. (*d*) After propagating 22.5 m after the VLS grating, σ_*y*_ and σ_*t*_ are 643 µm and 64.3 fs. (*e*) At the focus, the projection intensity profiles in the *y* dimension estimated by *Shadow*, *SRW*, analytic method, **K**-matrix and 6D phase space ray-tracing (this work). (*f*) At the focus, the projection intensity profiles in the *x* dimension are estimated by *Shadow*, *SRW*, analytic method, **K**-matrix and 6D phase space ray-tracing (this work).

In Fig. 3 ▸, the 6D phase space ray-tracing simulation results of the planar VLS grating beamline are presented. Fig. 3 ▸(*a*) illustrates the intensity distribution before the planar VLS grating in the (*y*, *t*) domain. Fig. 3 ▸(*b*) presents the intensity distribution at a distance of 7.5 m downstream of the planar VLS grating, where we observe pulse front tilt. We also found that the pulse duration is stretched. Fig. 3 ▸(*c*) depicts the intensity distribution at the focus located 15 m downstream of the planar VLS grating, where the pulse front tilt effect has vanished. Fig. 3 ▸(*d*) displays the intensity distribution 7.5 m after the focus, where the pulse front tilt reappears with a tilt angle opposite to that in Fig. 3 ▸(*b*). The evolution of the pulse propagation process is consistent with the previous results (Hu *et al.*, 2023*b* ▸). For accuracy comparison, at the focus, the intensity distribution is projected into *x* and *y* dimensions by integration as shown in Fig. 3 ▸(*e*) and Fig. 3 ▸(*f*), respectively. The projected beam is estimated using *Shadow* (Welnak *et al.*, 1994 ▸), *SRW* (Chubar & Elleaume, 1998 ▸), the **K**-matrix method (Hu *et al.*, 2023*a* ▸), the analytic method (Hu *et al.*, 2023*b* ▸) and the 6D phase space ray-tracing method (this work). In Fig. 3 ▸(*e*), at the *y* dimension, our results are consistent with the **K**-matrix method and analytic method. For *SRW* and *Shadow*, the beam size is significantly smaller due to the single wavelength assumption. In Fig. 3 ▸(*f*), in the *x* dimension, this work exhibits a high degree of agreement with the other methods. This is because there is no dispersion occurring in the *x* dimension.

## 5. Application in the simulation of a FEL beamline

In this section, we apply the 6D phase space ray-tracing method to simulate FEL pulse propagation through the FEL-1 beamline from the source point to the SCI endstation at S^3^FEL. The FEL-1 is designed to operate in self-amplified spontaneous emission (SASE) mode, covering the photon energy range 400 eV to 1240 eV. There are three experimental endstations at FEL-1, including the Spectroscopy Coherent Imaging endstation (SIC), the Ambient-Pressure X-ray Photoelectron Spectroscopy endstation (AP-XPS) and the Resonant Soft X-ray Scattering endstation (RSS).

**Figure 4:**
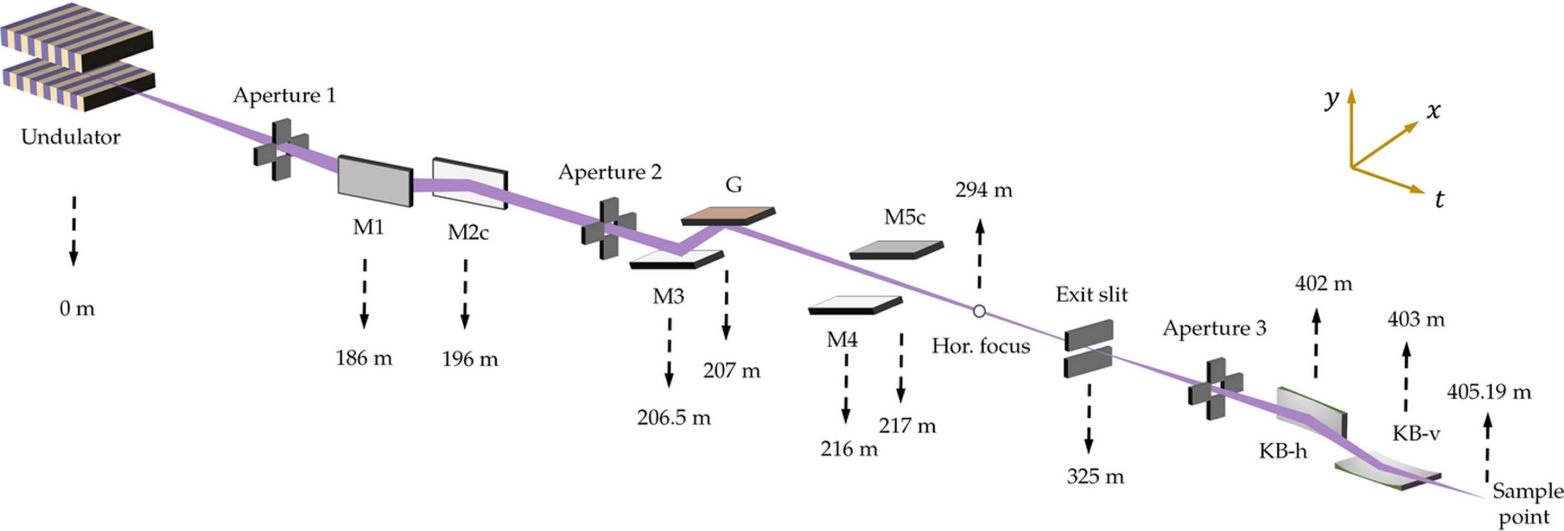
Preliminary optical layout of the SCI branchline of the FEL-1 beamline [reproduced from Zhu *et al.* (2024 ▸)]. The locations of the optics are marked by dashed arrows.

The preliminary optical layout of the SCI branchline of FEL-1 is shown in Fig. 4 ▸. Here, M1, M3 and M4 are plane mirrors, and M2c and M5c are cylindrical mirrors. G denotes the VLS grating. KB-h and KB-v are the horizontal and vertical mirrors of the KB system, respectively. The FEL-1 beamline uses a two-stage focusing strategy and switches between monochromatic and SASE modes by inserting or removing the grating monochromator (M3 and G) and M4-M5c from the optical path. In the first stage, M2c is used to generate a horizontal focus at a distance of 294 m, while M5c and G are used to produce a vertical focus at the exit slit. The exit slit extracts the monochromatic light from the input XFEL pulse diffracted by the grating. The second-stage focusing is done by the KB focusing system. The grating has a central groove density of 300 lines mm^−1^ and a VLS parameter of 2.8829 × 10^−5^ lines mm^−1^. Table 2 ▸ details the optical characteristics of the SCI FEL-1 beamline. θ_in_ and *R* represent the grazing incident angle and the radius of curvature of the mirror surface, respectively.

In the simulation, we first generate a 3D FEL optical field using *GENESIS*, then we convert it into a 6D geometric FEL source, and subsequently we simulate the monochromatic mode of the SCI FEL-1 beamline using the 6D phase space ray-tracing method. The FEL simulation parameters are summarized in Table 3 ▸.

**Figure 5:**
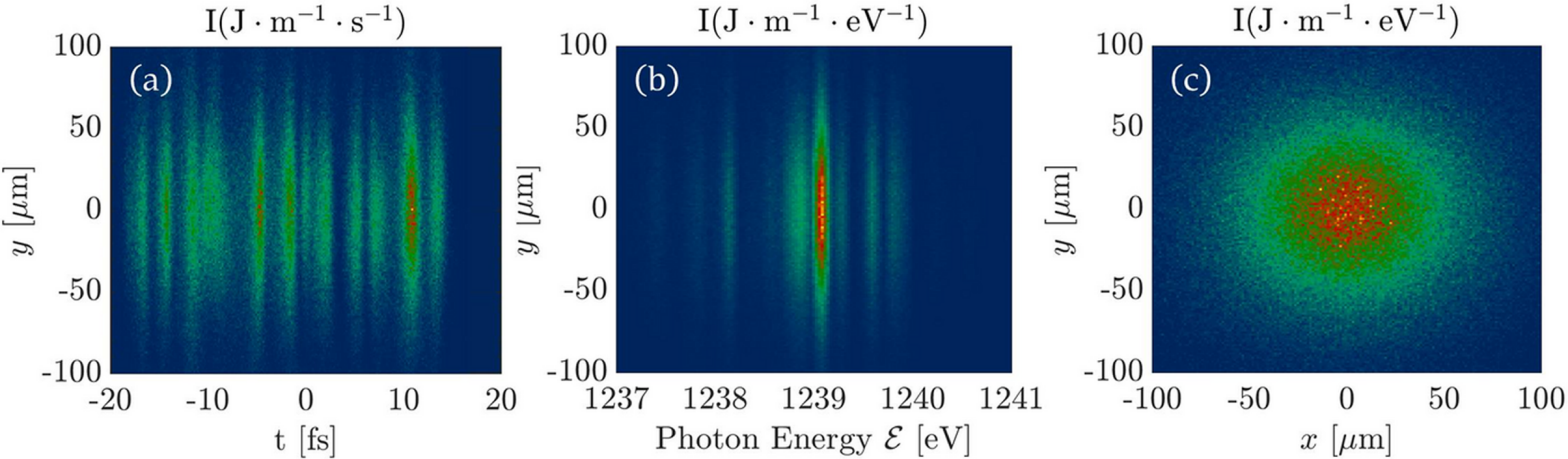
6D geometric FEL source. (*a*) Intensity distribution in the (*y*, *t*) domain. (*b*) Intensity distribution in the 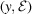 domain. (*c*) Intensity distribution in the (*x*, *y*) domain.

The 6D geometric FEL source is shown in Fig. 5 ▸. Fig. 5 ▸(*a*) represents the intensity distribution of the FEL pulse in the transverse–longitudinal (*y*, *t*) domain. Fig. 5 ▸(*b*) shows the intensity distribution of the FEL pulse in the transverse-spectrum 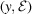 domain, and we can observe multiple spikes of the SASE spectrum. Fig. 5 ▸(*c*) illustrates the intensity distribution of the FEL pulse in (*x*, *y*) space, and we can find that the FEL pulse is close to a Gaussian distribution in the transverse domain.

**Figure 6:**
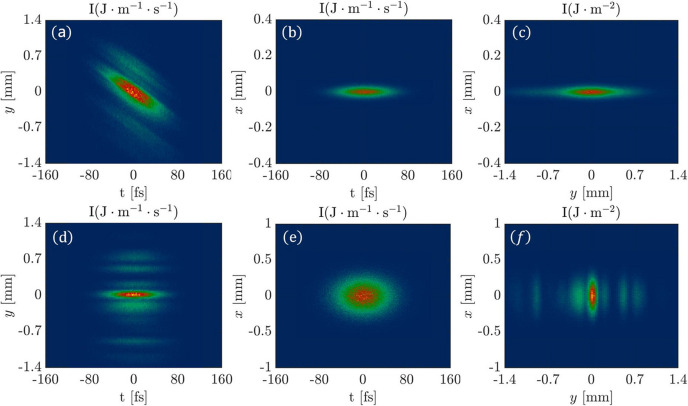
Snapshots of a FEL pulse propagation in the SCI FEL-1 beamline system (monochromator operation mode) at the horizontal focus and the exit slit. Panels (*a*), (*b*) and (*c*) illustrate the intensity distribution at the horizontal focus. Panels (*d*), (*e*) and (*f*) show the intensity distribution at the exit slit.

In the following, we perform a start-to-end simulation for the monochromatic mode of the SCI FEL-1 beamline using the 6D phase space ray-tracing method. The FEL pulse first passes through the off-set mirrors (M1 and M2c), then through the grating monochromator (M3 and G), followed by the exit slit and finally focused to the sample point by the KB mirrors. The simulation results are displayed at four locations: the horizontal focus, before the exit slit, 20 m after the slit and the sample point. Figs. 6 ▸(*a*), 6 ▸(*b*) and 6 ▸(*c*) represent the intensity distributions at horizontal focus in the (*y*, *t*), (*x*, *t*) and (*x*, *y*) domains, respectively. The simulation results indicate that the pulse front tilt is produced after the grating in the (*y*, *t*) domain, whereas in the (*x*, *t*) and (*x*, *y*) domains the intensity distributions resemble a Gaussian distribution. It is obvious that the FEL pulse duration is stretched after passing through the grating.

Figs. 6 ▸(*d*), 6 ▸(*e*) and 6 ▸(*f*) show the intensity distributions before the exit slit in the (*y*, *t*), (*x*, *t*) and (*x*, *y*) domains, respectively. We can observe that the pulse front tilt disappears before the exit slit. This indicates that the FEL pulse tilt angle changes with the propagation distance after passing through the VLS grating, and the tilt angle is zero at the focus (exit slit) where the intensity distribution in the *y* dimension represents the spectrum of the FEL pulse. Therefore, the exit slit can be utilized for filtering at the focus, which results in the monochromatization of the FEL pulse.

It is well known that diffraction occurs when light goes through a slit, and the diffraction effect cannot be described by geometric optics. In order to phenomenologically describe the diffraction effects in the 6D phase space ray-tracing method, we redistribute the divergence of the rays based on the Fraunhofer diffraction model. In the case of FEL-1 beamline at S^3^FEL, the diffraction effect occurs in the *y* dimension after the FEL pulse passing through the exit slit. In the following, we simulate the propagation of the FEL pulse after it has been monochromated by passing through the exit slit. We compare the 6D phase space ray-tracing method without diffraction correction, the 6D phase space ray-tracing method with diffraction correction and the Fourier optics based 3D pulse propagation method of *FURION*.

**Figure 7:**
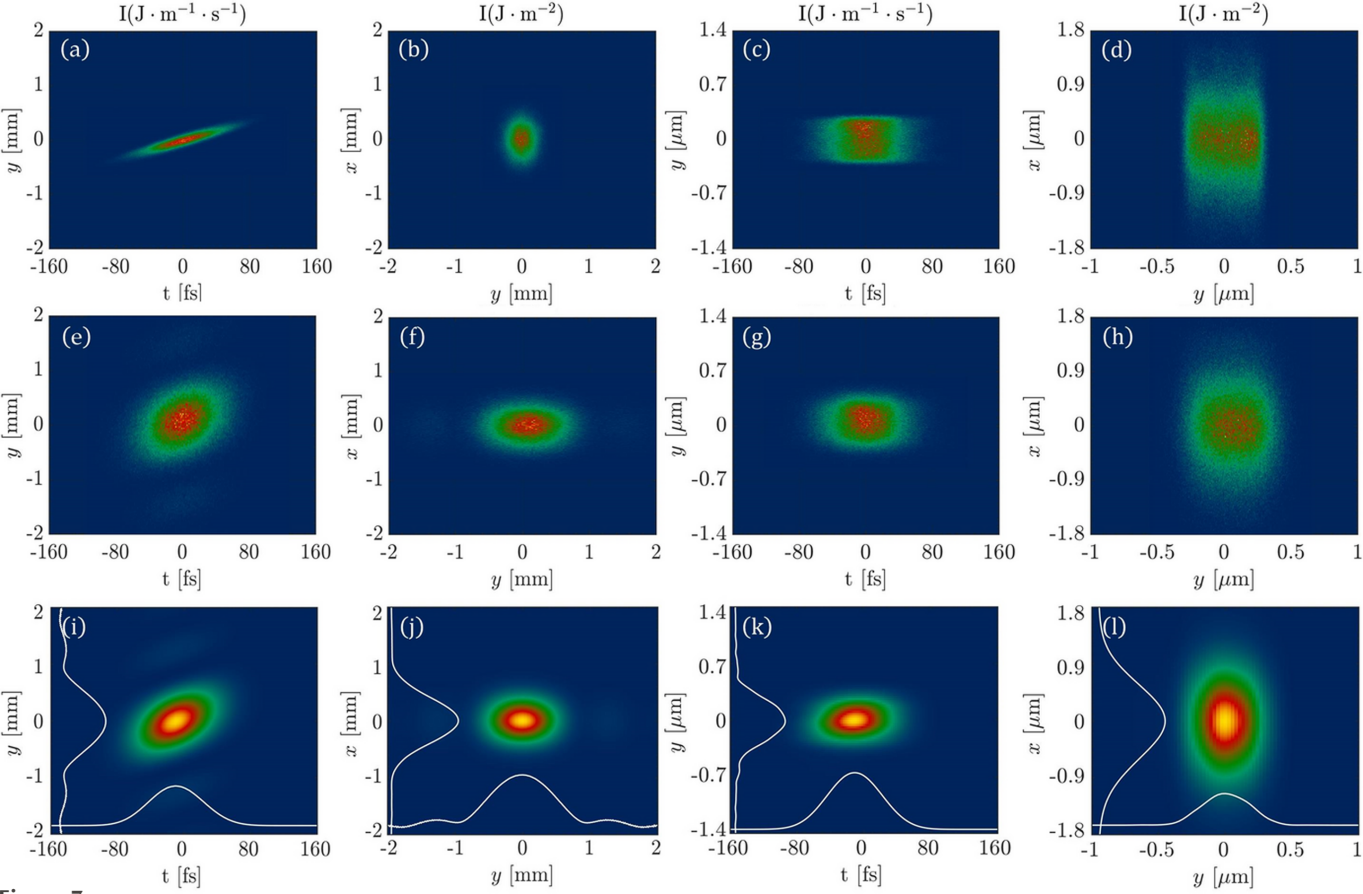
Snapshots of a FEL pulse propagation in the SCI FEL-1 beamline system (monochromator operation mode) at the position of 20 m after the slit and the sample point. (*a*), (*b*), (*c*) and (*d*) correspond to the results without diffraction correction. (*e*), (*f*), (*g*) and (*h*) show the results with diffraction correction. (*i*), (*j*), (*k*) and (*l*) are the results calculated by 3D pulse propagation module of *FURION*.

The top row of Fig. 7 ▸ represents the simulation results without diffraction correction. Figs. 7 ▸(*a*) and 7 ▸(*b*) represent the intensity distributions at a location 20 m downstream of the exit slit in the (*y*, *t*) and (*x*, *y*) domains, respectively. Figs. 7 ▸(*c*) and 7 ▸(*d*) represent the intensity distributions at the sample point in the (*y*, *t*) and (*x*, *y*) domains, respectively. The middle row of Fig. 7 ▸ shows the calculation results after applying diffraction correction. Figs. 7 ▸(*e*), 7 ▸(*f*), 7 ▸(*g*) and 7 ▸(*h*) correspond to the simulation results for the same locations and domains as those in Fig. 7 ▸(*a*), 7 ▸(*b*), 7 ▸(*c*) and 7 ▸(*d*), respectively. The bottom row of Fig. 7 ▸ displays the simulation results using the Fourier optics based 3D pulse propagation module of *FURION* (Zhu *et al.*, 2024 ▸). The simulation results indicate that the 6D phase space ray-tracing method with diffraction correction closely matches the simulation results of the 3D pulse propagation method based on Fourier optics.

In the FEL-1 beamline simulation, the 6D phase space ray-tracing method significantly reduces the simulation time compared with the 3D pulse propagation method (251 × 251 × 1199 source grid takes several minutes). The ray-tracing method achieves a time consumption of 15 s using 3 × 10^5^ rays. In these simulations, the CPU of the computer is 11th Gen Intel(R) Core(TM) i7-11800 H at 2.30 GHz. The speed advantage of the 6D phase space ray-tracing method over the pulse propagation method based on Fourier optics offers several practical benefits. In the beamline design stage, it enables researchers to conduct parameter sweeps and optimizations more efficiently. Moreover, when dealing with high-resolution calculations, such as strong focusing cases, the pulse propagation method may be limited by computational resources and time, making it difficult to achieve simulations. Our fast code can complete simulations within a reasonable time frame.

## 6. Summary

We developed a 6D phase space ray-tracing method for simulating X-ray beamline systems of synchrotron radiation light sources and FELs. This method can not only be used for simulating non-dispersive beamline systems but also for evaluating dispersive beamline systems. Compared with the Fourier optics based 3D pulse propagation approach, this method significantly reduces simulation time. We extended the hybrid method to the 6D phase space ray-tracing approach to describe the diffraction effect of limited aperture optics. Using this method, we can estimate the spatiotemporal coupling effects induced by dispersive X-ray optics, such as pulse front tilt, pulse front rotation, pulse compression and pulse stretching. We applied this method to simulate the SCI branchline of FEL-1 beamline at S^3^FEL; the simulation results show great agreement with that of the Fourier optics based 3D pulse propagation approach. This work can be used for the start-to-end simulation of beamlines, providing significant assistance in the design and optimization of beamlines. We have used this model in the *FURION* software package.

## Figures and Tables

**Table 1:** Source and grating parameters

Photon energy	413 eV
Beam size (RMS)	7.56 µm
Pulse duration (RMS)	14.86 fs
Bandwidth (RMS)	0.022 eV
Divergence (RMS)	31.58 µrad
Groove density	150 lines mm^−1^
VLS parameter, *b*_2_	3.714 × 10^−4^ lines mm^−1^
Incident angle, α	88.124°
Diffraction angle, β	87.450°

**Table 2:** Optics specifications of SCI FEL-1

Optics	Figure	θ_in_ (mrad)	*R* (m)
M1	Flat	12	–
M2c	Cylindrical	12	10889.2
M3	Flat	Scanning	–
M4	Flat	14	–
M5c	Cylindrical	14	10301.9
G	Flat	Scanning	–
KB-h	Bendable	17	–
KB-v	Bendable	17	–

**Table 3:** Simulation parameters of *GENESIS*

Electron energy	2.5 GeV
Undulator period	3 cm
Energy spread	0.4 MeV
Pulse duration	40 fs
Peak current	800 A
Average beta function	10 m
Photon energy	1240 eV
Normalized emittance	0.375 mm mrad
FEL parameter	8.9 × 10^−4^
Undulator parameter	1.0915
